# Effect of maternal birth positions on duration of second stage of labor: systematic review and meta-analysis

**DOI:** 10.1186/s12884-019-2620-0

**Published:** 2019-12-04

**Authors:** Marta Berta, Helena Lindgren, Kyllike Christensson, Sollomon Mekonnen, Mulat Adefris

**Affiliations:** 1 Department of Reproductive and Women’s Health, School of Midwifery, College of Medicine and Health Science, University of Gondar, Gondar, Ethiopia; 2 Department of Women’s and Children’s Health, Karolinska Institute, Solna, Sweden; 3 Institute of Public Health, College of Medicine and Health Science, University of Gondar, Gondar, Ethiopia; 4 Department of Gynecology and Obstetrics, School of Medicine, College of Medicine and Health Science, University of Gondar, Gondar, Ethiopia

**Keywords:** Maternal position, Flexible sacrum position, Second stage duration

## Abstract

**Background:**

It is believed that giving birth in an upright position is beneficial for both mother and the infant for several physiologic reasons. An upright positioning helps the uterus to contract more strongly and efficiently, the baby gets in a better position and thus can pass through the pelvis faster. Upright and lateral positions enables flexibility in the pelvis and facilitates the extension of the outlet. Before implementing a change in birthing positions in our clinics we need to review evidences available and context valid related to duration of second stage of labor and birthing positions. Therefore this review aimed to examine the effect of maternal flexible sacrum birth position on duration of second stage of labor.

**Method:**

The research searched articles using bibliographical Databases: Medline/PUBMED, SCOPUS, Google scholar and Google. All study designs were considered while investigating the impact of maternal flexible sacrum birthing positioning in relation duration of second stage of labor. Studies including laboring mothers with normal labor and delivery. A total of 1985 women were included in the reviewed studies. We included both qualitative and quantitative analysis.

**Results:**

We identified 1680 potential citations, of which 8 articles assessed the effect of maternal upright birth positioning on the reduction during the duration of second stage of labor. Two studies were excluded because of incomplete reports for meta analysis. The result suggested a reduction in duration of second stage of labor among women in a flexible sacrum birthing position, with a mean duration from 3.2–34.8. The pooled weighted mean difference with random effect model was 21.118(CI: 11.839–30.396) minutes, with the same significant heterogeneity between the studies (I^2^ = 96.8%, *p* < 000).

**Conclusion:**

The second stage duration was reduced in cases of a flexible sacrum birthing position. Even though the reduction in duration varies across studies with considerable heterogeneity, laboring women should be encouraged to choose her comfortable birth position. Researchers who aim to compare different birthing positions should consider study designs which enable women to choose birthing position.

**Prospero registration number:**

[CRD42019120618]

## Background

The second stage of labor begins when the cervix is completely dilated (open) and ends with the birth of the baby. In research, the second stage is often divided into a passive phase, an active phase, and the actual birth of the baby when the baby actually emerges [1]. Giving birth in an upright position can benefit the mother and baby for several physiologic reasons [2]. When a laboring woman is in upright position to give birth, there is less risk of compressing the mother’s aorta, which means there is a better oxygen supply to the baby [3]. Upright positioning also helps the uterus contract more strongly and efficiently as a result it helps the baby get in a better position [2, 4].

In summary, the purpose of implementation of an upright position is for the enhancement of uterine contractions, fetal condition, and the promotion of maternal comfort [5–7]. Flexible sacrum positions (FSP = knee-standing, on all fours, sitting on a birth seat and lateral) is where weight is taken off the sacrum, thereby allowing the pelvic outlet to expand well [8, 9].

A Cochrane review examined duration of the second stage of labour, comparing limited birth positions (upright, birth-stool/squatting and birth chair/cushion) with supine/lithotomy positions, excluding water birth, mothers without epidural anesthesia and studies from low income countries. An update on this review was done in 2017 [10, 11]. In our present study we take into account all studies incorporating the above mentioned birthing positions (FSP), from all settings, observational and experimental studies and year of publication. Even though the issue has frequently been studied; evidence related to alternative birthing positions is not well known. Among all clinical midwives, this knowledge helps midwives to encourage laboring women and their families to make informed decisions regarding positions to be used in childbirth [3]. In order for midwives to optimize their care for laboring women, there is a need for evidence to support and advocate for women during the labor and delivery process. Thus, systematic review and meta-analysis with the objective of assessing the effect of maternal flexible sacrum birthing positions on duration of the second stage of labor was conducted.

### Objective

To determine the effect of maternal flexible sacrum birthing positions on duration of second stage of labor in comparison with supine position.

## Methods

### Eligibility criteria

Any cross sectional, observational, cohort studies and RCT studies comparing flexible sacrum (standing, kneeling, sitting, squatting and birthing ball and lateral positions) against supine position, were peer-reviewed and reported in original research articles were considered for the present review.

All pregnant women with normal labor at health facility, the main comparison was the use of any upright or lateral position during the second stage of labor (FSP) compared with supine or lithotomy/recumbent/semi-recumbent positions.

The primary outcome is duration of second stage of labor. No secondary outcome was taken in to consideration.

We excluded studies reported in languages other than English, systematic review and meta analysis, studies considering high risk pregnancy and inaccessible full-text articles.

### Search strategy

Data base (www.crd.york.ac.uk/prospero) was explored to confirm whether systematic review or meta-analysis existed before. The titles of all appropriate abstracts and titles collected from electronic and manual searches were entered into the EndNote-7 reference software. The reference lists of all the articles were also scrutinized for further studies.

Potentially relevant articles for the review were identified by searching bibliographical Databases: Medline/PUBMED, JBI library and SCOPUS. Google scholar and Google were searched to include all pre-reviewed articles. Search terms used were directly related to the title: women, labor second stage, upright position, duration, supine position and birth. In the search strategy we included combination of keywords extracted from the title: effect Or influence AND maternal OR women AND positions (standing, kneeling, all four, sitting, squatting, lateral, supine) AND birth OR delivery OR parturition AND duration AND second stage of labor. Additional relevant articles were identified by searching the reference lists of full-text articles and grey literatures from Google and Google scholar.

### Study selection

Each title and abstract was screened by two independent reviewers using a standardized form [12]. Each full text article was reviewed by two independent reviewers using standardized inclusion criteria: (a) presents primary data analysis; (b) uses a quantitative method of data collection and analysis (quantitative studies); (c) discusses maternal birth position in relation to duration of second stage; (d) discusses childbirth occurring in health facilities; and (e) was published in English. Discrepancies during title and abstract and full text screening were resolved by discussion with a third reviewer until consensus was reached.

### Quality assessment

All papers selected for inclusion were subjected to a rigorous, independent appraisal by the investigators using standardized critical appraisal instruments adopted from JBI. The tool addresses both the external and internal validity and has multiple items for each type of study for risk of bias. Furthermore, it has nine items for cross-sectional and thirteen items for RCT to be used. The overall risk of study bias ranked into one of the four levels (High, Moderate, Low, Very Low), for inclusion or exclusion of studies. The reviewers for this study interpret this ranking system based on the recommendation from JBI reviewer manual, (High = 75–100%, Moderate = 50–75%, Low = 25–50 and < 25%). Hence we decided to include studies which score with high (75–100%) and moderate (50–75%). Accordingly, only one paper lies in the moderate range and the others seven lie in the high range [13].

To ascertain scientific rigor, we used the Preferred Reporting of Systematic Reviews and Meta-Analysis (PRISMA) guidelines for systematic data analysis [14]. The two reviewers were blinded to each other for screening of studies, data extraction, and risk of bias assessment parts of the review. If any differences seen when we compare results from the two reviewers, the third reviewer was communicated.

### Data extraction and outcome of interest

Data were extracted from each study included in the review using a pre-constructed criteria based on the standardized JBI data extraction tool [15]. Two authors extract data and they compared the results; discrepancies were resolved by discussion by the reviewer made, for the decision third reviewer was contacted. We were contacted the original authors of the eligible studies through email or phone for further clarification of data. For each study we extracted the following domains.

i) Author(s) and years of publication
ii) Study designs (cross sectional, observational, cohort and RCT studies)
iii) Country or region
iv) Sample size for each groups
v) Main findings (mean and standard deviation of second stage duration in each group)

The outcome of interest was duration elapsed in the second stage of labor measured in minutes.

### Data analysis

We undertook an initial descriptive analysis of the studies. Heterogeneity between estimates was assessed using the I^2^ statistic, to describe the percentage of variation not because of sampling error across studies. An I^2^ values above 75% indicates considerable heterogeneity [16].

Potential influences on mean estimates was investigated using subgroup analyses, we compared mean estimates by region, within studies. Pooled mean difference of labor duration of FSP birthing positions versus supine position in the second stage was analyzed using statistical meta-analysis software STATA version11.

## Result

### The review process

**Fig. 1:**
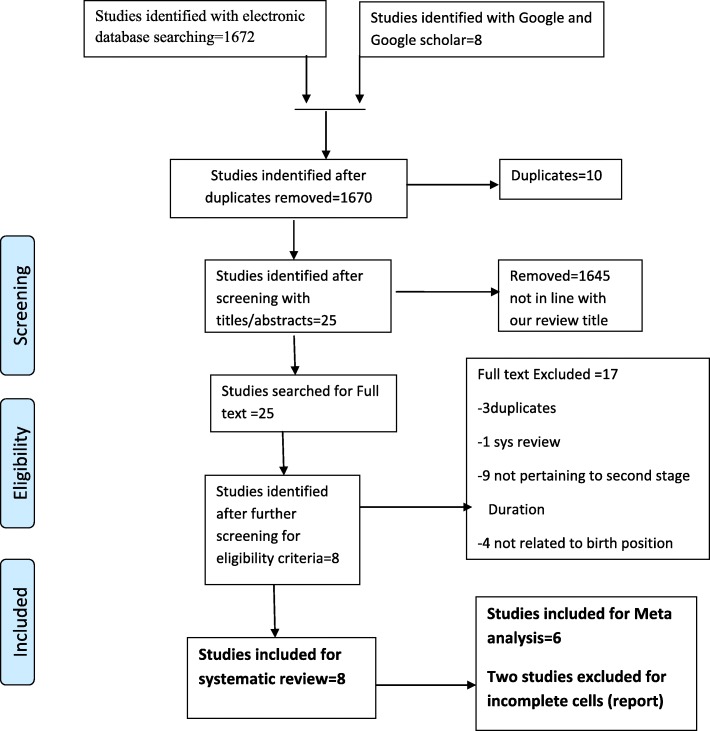
PRISMA Flow chart of search and study inclusion process

Over all we found 1680 studies with our search strategies. The initial search from PUBMED yielded 1660 studies, another search from SCOPUS yielded 12 studies and from manual search we get 8 studies making a total of 1680, of which 10 duplicates were removed. After title and abstract screening 1645 studies were excluded since they didn’t fulfill the inclusion criteria, 25 potentially relevant articles were searched for full text. Eight studies met the inclusion criteria and 17 studies were excluded. Of these 3 studies were duplicates, one study was a systematic review, 9studies were not related to birthing position and 4 were not pertaining to duration of second stage of labor. Finally, we synthesize 8 studies for systematic review and 6 studies for meta analysis (Fig. 1).

### Characteristics of included studies

The sample size from the 8 included studies with the total of 1985 laboring women (933 for supine position and 938 for flexible sacral position). As seen from Table 1, one of the studies was a cross-sectional study, 7 studies were RCT. One study was conducted in an African country, and three were done in India. The other four were done in high-income countries (Spain, Turkey, Finland and U.K).

**Table 1:** Presentation of the summary results of the included studies

Author, year and country	Study design	Total sample size	Positions in comparison	Results			Bias/ Limitation
				Mean (minutes) for Upright/lateral	Mean (minutes) for Supine	Length of time shortened by upright position	
Simaro M., 2017 (Spain)	RCT	155	All upright/lateral Vs supine	94.6	124.3	29.7	Low risk
Denakpo J., 2012 (Benrin)	CS	980	Standing, sitting and squatting Vs supine	159.5	179.3	19.8	Low risk
Gupta JK, 1989 (U.K)	RCT	114	Squatting Vs supine	36	40	4	Low risk
Mathew A., 2012(India)	RCT	60	Birthing ball & ambulation Vs supine	23.9	49.8	25.9	Low risk
Mraloglu O., 2017 (Turky)	RCT	100	Squatting Vs supine	21.02	55.4	34.38	Low risk
Dabral A., 2018 (India)	RCT	300	Kneeling Vs supine	23.9	39.38	15.48	Low risk
Marittila M., 1983 (Finland)	RCT	100	Sitting Vs supinr	21.8	25	3.2	Low risk
Thilagavathy G.,2012 India)	RCT	200	Half sitting Vs supine	56	67	11	Low risk

The difference in duration of second stage of labor from supine to FSP was high across the studies that reported all in minutes, ranging from 3.2 to 34.4 min. All the included studies were conducted in health facilities. Among the 8 included studies, two studies compare squatting position Vs supine [16, 17], two studies compare sitting position Vs supine, [18, 19], one compare keeling Vs supine [20], two studies compare flexible sacral position Vs supine [21, 22] and one study compare ambulation and birthing ball with supine position. Two studies allowed laboring women for free choice of birthing position [21, 22]. Two studies calculate minimum sample size using sample size calculation with the assumptions for double population [16, 22].

### Weighted mean difference of duration of second stage of labor

**Fig. 2:**
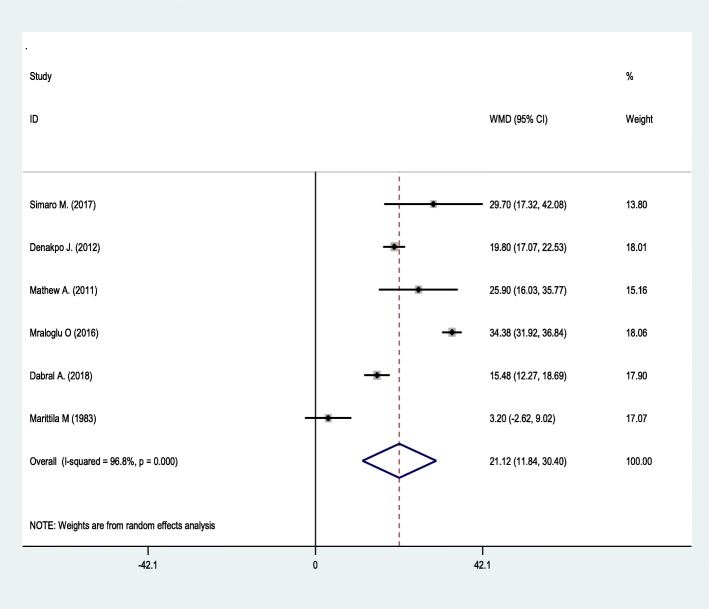
Duration of second stage with random effect model

In our meta analysis two studies were excluded [16, 19] for their incomplete report. The overall estimated mean difference of duration of second stage of labor from the included six studies with fixed effect model showed a significant heterogeneity between the studies. So that the main meta analysis was fitted to random effect model to get the pooled mean. The duration of second stage of labor across the studies included was ranges between 3.2–34.38 min. The pooled weighted mean difference with fixed effect model was 23.47 (95%CI: 21.96–24.97) minutes and with random effect model was 21.118(CI: 11.839–30.396) minutes, with the same significant heterogeneity between the studies (I^2^ = 96.8%; very low-quality evidence, *p* < 000) (Fig. 2).

#### Subgroup analysis

**Fig. 3:**
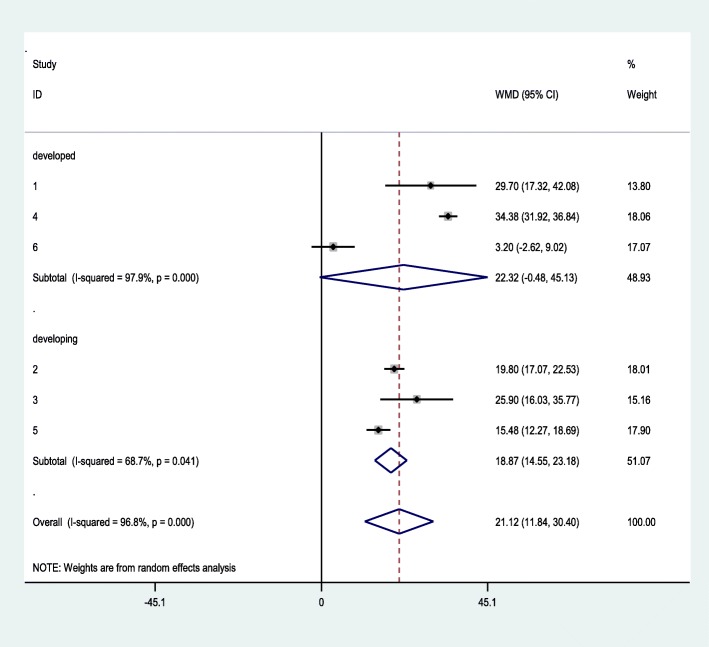
Sub-group analysis by region

Subgroup analysis was done based on region in order to identify the potential heterogeneity between studies. In this sub-group analysis studies were grouped in to low-middle and high income regions to see the effect on heterogeneity. The sub-total weighted mean difference of duration of second stage of labor was higher in high income region across studies as compared to low-middle income region. Hence studies conducted in low-middle income regions showed significant improvement in heterogeneity (18.87, 95% CI: 14.55–23.18, I^2:^ 68.7%, *P* < 0.041), as compared to the developed region (22.32, 95% CI: − 0.48-45.13, I^2^: 97.9%, *P* < 0.000) as shown in Fig. 3.

### Sensitivity analysis

**Fig. 4:**
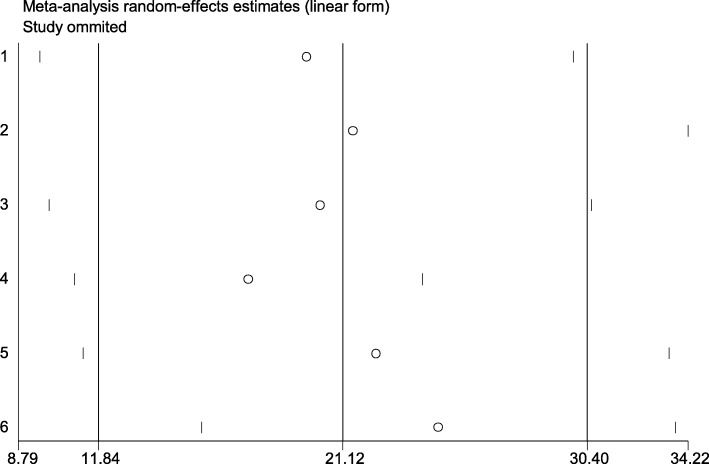
Sensitivity analysis

The effect of an individual study for causing the heterogeneity was conducted, but no any influential study was identified since all studies were within the confidence interval. Thus, no further analysis for sensitivity was needed (Fig. 4).

### Assessment of publication bias

**Fig. 5:**
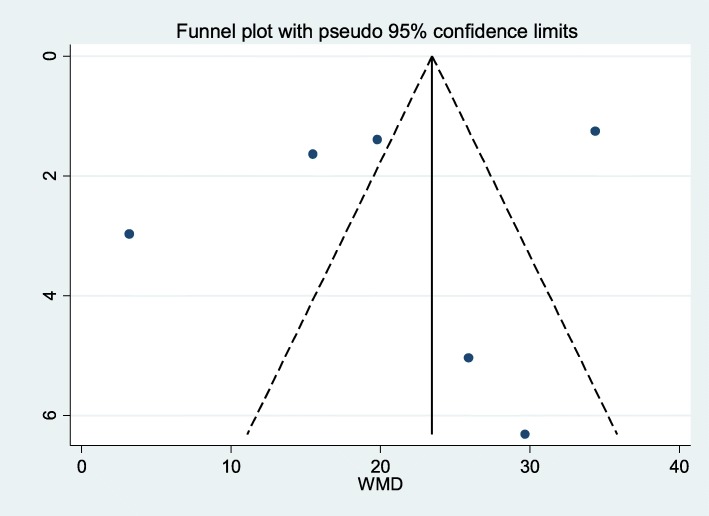
Presentation of funnel plot

Publication bias was assessed using Egger’s test. The estimated bias coefficient was − 2.14 (Egger bias B = − 2.14 (95% CI: − 7.03-2.75)) with a standard error of 1.76, giving a *p*-value of 0.291. Thus, the test provides no evidence for the presence of small-study effect. Figure 5 presents the funnel plot result with the 95% confidence limit.

## Discussion

The review showed that using a flexible sacrum position can reduce the duration of the second stage of labor by 21.12 min. The reduction was contributed mainly by a large reduction in the three studies of the birthing ball, flexible sacrum and squatting positions reduce 25.9, 29.7 and 34.38 min respectively [17, 22, 23]. The reduction in duration is in line with other review and meta-analysis conducted both in UK in different times, in contrast other meta-analysis done in Australia and UK, didn’t show any reduction in duration of second stage [10, 11]. This difference may be due to the variable trial quality, inconsistencies within trials (in different birth position) used in different period of time and in different settings and heterogeneity of participants in individual studies. The reduction in second stage duration have greater advantages for both the mother and her infant by decreasing unnecessary intervention for the mother and reduced fetal heart rate abnormality, neonatal hypoxia and acidosis [24] . In another way reduction in second stage of labor may cause both maternal and neonatal trauma due to fast expulsion of the fetal head [25].

The sub-group meta-analysis reported that an overall pooled mean difference in reduction of second stage of labor among the low-middle income regions was significant as compared to high-income region. Keeping the heterogeneity between the studies for the high-income region is highly considerable, thus it ends up with wide confidence interval and include non-significant value.

The reduction in duration of second stage of labor between two studies with same comparison (squatting Vs supine) showed high difference, ranges between 4 and 34.38 min [16, 17].

In the present review, we only found two studies where women in the intervention group could choose freely between the upright or lateral positions. One of the studies compared flexible sacrum position Vs supine, which resulted in a mean difference of 29.7 min [22]. Women used a minimum of two and a maximum 5types of flexible sacrum positions until they completed the labor and delivery [22]. The other study compared three upright positions (sitting, standing and squatting) Vs supine, this also results in remarkable reduction in duration (19.8 min) [21], but it didn’t compare the difference in reduction of duration of second stage of labor of each upright against supine. In these two studies women were allowed to freely choose between the upright or lateral positions. Having this opportunity to choose, might make women become relaxed and feel comfortable. It also might facilitate the rotation and descent of the baby’s head and hence contribute to the reduction in duration of second stage of labor [26].

### Limitation of this review

Our review uses limited data bases (PUBMED & SCOPUS) even though extensive search was done using these two data bases. We couldn’t however access other data bases because their sites are not accessible. There was a high variation in sample size, setting, and time between studies that may affect the quality of our review.

## Conclusion

Flexible sacrum birthing position has effect on reduction in duration of the second stage of labor with a considerable variation was reported. This reduction in duration of second stage of labor should be discussed among health care providers who care for women during labor and childbirth.

### Implications

Laboring women should be encouraged to choose a birth positions that she finds comfortable. Researchers who aim to compare different birth positions should consider study designs which enables women to choose birthing position.

## Data Availability

The datasets supporting the conclusions of this article are included within the article.

## Abbreviations

FSP: Flexible Sacrum Position
JBI: Joanna Briggs Institute
PRISMA: Preferred Reporting of Systematic Reviews and Meta-Analysis
RCT: Randomized Controlled Trial
U.K: United Kingdom
